# Appendiceal phlegmon in adults: Do we know how to manage it yet?

**DOI:** 10.1016/j.amsu.2020.08.033

**Published:** 2020-08-31

**Authors:** Pedram Panahi, Rashid Ibrahim, Pushpa Veeralakshmanan, James Ackah, Mark Coleman

**Affiliations:** Department of General Surgery, University Hospitals Plymouth NHS Trust, Derriford Road, Plymouth, PL6 8DH, United Kingdom

**Keywords:** Appendiceal mass, Appendicular/appendix mass, Appendiceal phlegmon, Appendicular/appendix phlegmon, Early appendicectomy, Interval appendicectomy, Conservative management, Evidence-based medicine

## Abstract

A Best Evidence Topic in general surgery was written according to a structured protocol. The question addressed was ‘Appendiceal phlegmon in adults: Do we know how to manage it yet?‘. Altogether 217 papers were found on Ovid Embase and Medline, 334 on PubMed and 13 on the Cochrane database using the reported search. From the screened articles, 5 represented the best evidence to answer the clinical question. The authors, journal, date and country of publication, patient group studied, study type, relevant outcomes and results of these papers are tabulated. We conclude that the best management method is conservative only treatment without interval appendicectomy. These patients must be followed up, including colonoscopy and/or CT imaging as indicated, to investigate for conditions such as inflammatory bowel disease or malignancy masquerading as appendicitis.

## Highlights

• On-going controversy exists around the best management option for an appendiceal phlegmon.
• Various articles advocate each of the following: early appendicectomy, interval appendicectomy and conservative management.
• Therefore, we conducted a literature review in the format of a Best Evidence Topic to identify the best approach.
• We conclude that the best management method is conservative only treatment without interval appendicectomy.
• They must be followed up, including colonoscopy/imaging as indicated, to identify conditions masquerading as appendicitis.
• Our suggestion is in direct contrast to the widely practiced method of interval appendicectomy.

## 1 Introduction

A Best Evidence Topic was constructed according to a structured protocol. This is fully described by the International Journal of Surgery [1].

## 2 Clinical scenario

A 29-year-old male patient presents with a 7-day history of migratory central to right lower quadrant abdominal pain. This is associated with nausea, anorexia and low-grade pyrexia. On examination, a mass like structure is palpated in the right lower quadrant. Ultrasound scanning confirms your diagnosis of an appendiceal mass. You recall the ongoing controversy around the best management options: early appendicectomy, interval appendicectomy and purely conservative management. Unsure which is in the best interest of the patient, you resolve to check the literature for evidence.

## 3 Three-part question

In [adult patients with an appendiceal phlegmon], what is the best management option out of [early appendicectomy, interval appendicectomy and purely conservative management] in terms of [length of admission, complications and other clinical outcomes]?

## 4 Search strategy

The search strategy outlined below was utilised and where possible the results were limited to English articles, human studies and adult population. In addition, the reference lists of the screened articles were reviewed.

Medline 1946 to May 2020 and Embase 1974 to May 2020 using the OVID interface:

[Appendiceal mass OR appendicular mass OR appendix mass OR Appendiceal phlegmon OR appendicular phlegmon OR appendix phlegmon] AND [appendicectomy OR appendectomy]

Medline using the PubMed interface:

[Appendiceal mass OR appendicular mass OR appendix mass OR Appendiceal phlegmon OR appendicular phlegmon OR appendix phlegmon] AND [appendicectomy OR appendectomy].

### 4.1 Search outcome

**Fig. 1:**
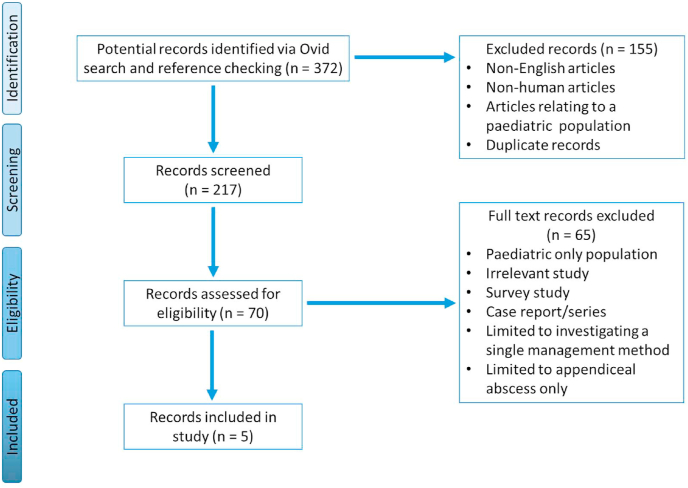
*PRISMA flow chart for Ovid search (PubMed and Cochrane flow chart not included in this figure)*.

217 papers on Ovid Embase and Ovid Medline, 334 papers on PubMed and 13 papers on the Cochrane database were found using the reported search and screened. From these 5 papers were identified that provided the best evidence to answer the question of the optimal management method for an appendiceal phlegmon out of early appendicectomy (EA), interval appendicectomy (IA) and purely conservative management (CM). These are presented in Appendix 1. An example of the screening and eligibility assessment process for the search results obtained from the Ovid interface is detailed in Fig. 1.

## 5 Results

The results of this article are tabulated in Appendix 1; this contains a review of the most relevant and highest quality evidence available assessing the optimal method of appendiceal mass management. This table is structured according to the guidance by the International Journal of Surgery [1] highlighting key results, statistical analysis outcomes and study limitations.

## 6 Discussion

The management of an appendiceal mass is an area of ongoing debate and controversy; a recent survey study by Sajid et al. displayed the immense disparity in the perceived optimal management method amongst general surgeons [2]. Considering this, we conducted a review of the Best Evidence Topic.

Demetrashvili et al. [3] performed a prospective study comparing EA, IA and CM. Their study favoured the approach of conservative treatment with antibiotics and image-guided drainage where indicated, without the need for IA unless there was a recurrence of appendicitis. Of interest was the finding that of the 3 patients in the CM group who developed recurrence of appendicitis, all had undergone percutaneous drainage as part of their conservative management. Following completion of conservative management, Colonoscopy and CT imaging was recommended within 4–6 weeks; this approach is justified in obviating the considerable risk of missing hidden pathologies associated with CM [4].

Aranda-Narváez et al. [5] conducted a retrospective study with a control group of EA matched to a CM/IA group. An initial conservative management was successful in all cases without requirement for urgent surgical intervention. Only 13.3% of the laparoscopic approach to the EA was completed without conversion to open, though it is important to note that the data sampling was done from 1997 to 2009 whereby advances in minimally invasive surgery were ongoing [6]. This study, similarly to that of Demetrashvili et al., concluded that the best method of management is that of a purely conservative management, only with the addition of IA in cases of symptomatic recurrence. The mean percentage of recurrence across the tabulated articles of Appendix 1 was 17%; this is comparable to the figures quoted by other studies, which range from 12% to 20% [[7], [8], [9]].

Cheng at al [10] conducted a Cochrane systematic review in 2017 of randomised controlled trials (RCT) comparing EA and IA, and identified only two suitable RCTs. It is important to note that the reviewers regarded both trials as very low quality trials. Further, this Cochrane review did not have a meta-analysis due to the fact that only one trial was identified for each outcome (appendiceal phlegmon and appendiceal abscess). In our article, it was decided to include only one of the two RCTs in the results table due to the fact that the other RCT's patient population consisted of only paediatric patients with appendiceal abscesses. The included RCT is that by Kumar et al. [11] who concluded that the best management modality was again of CM without appendicectomy. Of note is the fact that this trial does not investigate quality of life.

In 2014, Olsen et al. [12] conducted a systematic review of studies investigating treatment modalities of CM and EA, but not of IA. The results included in the table are specifically that of the adult population, but not of the paediatric or mixed population studies. In adults, it was identified that the frequency of complication in EA ranged between 0 and 57%: major complications occurred in up to 19% and the risk of intestinal resection was approximately 10%. On the other hand, this systematic review mentions that all of the patients with a phlegmon responded to antibiotics. Overall, Olsen et al. concluded that CM is the safest method of appendiceal mass management, with a low risk of treatment failure and complications.

Due to the fact that the aforementioned study did not investigate IA, it was decided to include the systematic review by Darwazeh et al. [13] comparing CM and IA. It is important to note that the number of CM studies included in this review were 21 which was considerably higher compared to the IA group which comprised of 5 studies. The commonest morbidity in the CM group was unresolved sepsis requiring surgery. The commonest morbidities in the IA group were infections and bowel obstruction. A particular weakness of the study to highlight is the fact that the cumulative days of hospital admission and morbidity were calculated using a simple addition of the values from the IA group to the CM group; this may provide an inaccurate number, particularly if the IA studies themselves had already included both initial admission and re-admission. Despite this, they concluded that IA is of minimal benefit, whereby the number needed to treat to prevent one case of recurrence is 8.

Overall, it is clear from the discussed articles that conservative management with appropriate follow up is the best management method. The average of data from the tabulated studies comparing EA to IA indicated that the operative time of EA was 22.8 min longer, the admission length of EA was 4.5 days longer and the complication rate of EA was 40.9% higher. Therefore, if surgery was indicated, IA is a superior option to EA.

## 7 Clinical bottom line

In the management of an appendiceal phlegmon in an adult, it is evident from the discussed articles that the best management method is conservative only treatment without interval appendicectomy. These patients must be followed up, including colonoscopy and/or CT imaging as indicated, to investigate for conditions such as inflammatory bowel disease or malignancy masquerading as appendicitis.

Our suggestion is in direct contrast to the widely practiced method of interval appendicectomy [14], popularised as the Ochsner-Sherren regimen. It is important to note that to this day there are no prospective, large-scale and well-designed clinical trials with blinding of outcome assessors directly comparing CM, EA and IA; this highlights an important area for future research.

## Declaration of competing interest

None to declare.

## Figures and Tables

**Table:** 

Article	Author, date, journal, country and study type (Level of Evidence)	Patient group	Outcomes	Key results	Comments
Comparison of treatment methods of appendiceal mass and abscess: A prospective Cohort Study [3]	Demetrashvili et al. (2019), Annals of Medicine and Surgery, Georgia	N = 74 patients	Operative time	EA (117.7 min) vs IA (87.1 min) [P = 0.008]	Does not differentiate between phlegmon and abscess. Small Sample size. Not randomised. Excluded immunocompromised and ASA 4–5 patients.
			Total length of admission	EA (10.4 days) vs IA (8.1 days) [P = 0.009]	
	Prospective cohort study (Level III)		Complications	EA (22.2%) vs IA (0%) [P = 0.02]	
			Other	In EA group, ileocecectomy/right hemicolectomy performed in 25.9% of cases compared with 8.33% in IA group. Appendicitis recurred in 13% of the CM group who proceeded with appendicectomy with no complications post-operatively. In this group, both operative time (51.4 min) and hospital stay (4.3 days) was significantly shorter compared to IA group. 3 patients (4.05%) had hidden pathologies of malignancy and Crohn's disease.	
Conservative approach versus urgent appendectomy in surgical management of acute appendicitis with abscess or phlegmon [5]	Aranda-Narváez et al. (2010), Revista Española de Enfermedades Digestivas, Spain	N = 30 patients	Operative time	No statistically significant difference between EA and IA.	Retrospective study. Small sample size. Patients with severe sepsis excluded.
			Total length of admission	No statistically significant difference.	
	Retrospective cohort study (Level III)		Complications	Contaminated surgery: EA (100%) vs IA (28.6%) [P < 0.001]. Surgical site infection: EA (40%) vs IA (0%) [P < 0.001]	
			Other	Appendicitis recurred in 33.3% of the CM group who proceeded with appendicectomy with no complications post-operatively. 1 patient (3.33%) had hidden pathology of caecal adenocarcinoma.	
Early versus delayed appendicecto-my for appendiceal phlegmon or abscess (Review) [10]	Cheng et al. (2017), Cochrane Database of Systematic Reviews, China	N = 40 patients (Only one of the two studied RCTs included here as the other RCT was purely based on appendiceal abscesses in a paediatric population)	Operative time	EA (53 min) vs IA (38 min) [Extracted from original RCT article rather than Cochrane review. P < 0.05]	No pooling of data/meta-analysis. No power calculation, small sample size and recruitment of a mixture of both paediatric and adult patients in the included RCT. Included RCT at high risk of performance and reporting bias, and unclear risk of selection bias (No blinding, unclear method of randomisation and allocation concealment). Sampling done between 1998 and 2001 consisting of only open appendicecto-my.
			Total length of admission	EA (21.4 days) vs IA (14.7 days) [P < 0.001]. NB original article indicates 4.9 days length of admission in CM group.	
	Systematic review of 2 RCTs (Level I)		Complications	Complication rate (Wound infection and faecal fistula): EA (30%) vs IA (0%) [Risk Ratio 13, P = 0.07]	
			Other	Time away from normal activities: EA (25 days) vs IA (20 days) [P = 0.005) No cross over from IA group to EA group. 2/20 (10%) patients in CM group had recurrent appendicitis requiring appendicectomy.	
Treatment of appendiceal mass – a qualitative systematic review [12]	Olsen et al. (2014), Danish Medical Journal, Denmark	N = 48 studies (Only data for adult population studies included here)	Total length of admission	Noted but not assessed	Does not investigate IA. Includes both phlegmon and abscess. No meta-analysis. Studies ranged from 1969 to 2013.
			Treatment failure	CM (0–22%)	
	Systematic review (Level I)		Complications	EA (Range of 0–57%)	
			Other	Studies on recurrent appendicitis excluded.	
A Systematic Review of Perforated Appendicitis and Phlegmon: Interval Appendectomy or Wait-and-See? [13]	Darwazeh et al. (2016), The American Surgeon, United States	N = 1943 patients (26 studies)	Operative time	Not assessed.	Does not investigate EA. Includes both phlegmon and abscess. Mixture of paediatric and adult patients. No meta-analysis.
			Total length of admission	CM group (9.6 days) vs IA (14.6 days - Cumulative estimate including initial admission) [Significance not assessed]	
	Systematic review (Level I)		Complications	CM group (13.3%) vs IA (23.7% - Cumulative estimate including initial admission) [Significance not assessed]	
			Other	12.4% recurrence of appendicitis in CM group.	
